# Development of a daily predictive model for the exacerbation of chronic obstructive pulmonary disease

**DOI:** 10.1038/s41598-023-45835-4

**Published:** 2023-10-31

**Authors:** Yong Suk Jo, Solji Han, Daeun Lee, Kyung Hoon Min, Seoung Ju Park, Hyoung Kyu Yoon, Won-Yeon Lee, Kwang Ha Yoo, Ki-Suck Jung, Chin Kook Rhee

**Affiliations:** 1 Division of Pulmonary and Critical Care Medicine, Department of Internal Medicine, College of Medicine, Seoul St. Mary’s Hospital, The Catholic University of Korea, 222 Banpo-daero, Seocho-Gu, Seoul, 06591 Republic of Korea; 2 Department of Statistics and Data Science, Yonsei University, Seoul, Republic of Korea; 3 Department of Applied Statistics, Yonsei University, Seoul, Republic of Korea; 4 Division of Pulmonary, Allergy and Critical Care Medicine, Department of Internal Medicine, Korea University Guro Hospital, Korea University College of Medicine, Seoul, Republic of Korea; 5 Department of Internal Medicine, Jeonbuk National University Medical School, Jeonju, Republic of Korea; 6 Division of Pulmonary and Critical Care Medicine, Department of Internal Medicine, College of Medicine, Yeouido St Mary’s Hospital, The Catholic University of Korea, Seoul, Republic of Korea; 7 Department of Internal Medicine, Yonsei University Wonju College of Medicine, Wonju, Gangwon Republic of Korea; 8 Division of Pulmonary and Allergy Medicine, Department of Internal Medicine, Konkuk University School of Medicine, Seoul, Republic of Korea; 9 Division of Pulmonary Medicine, Department of Internal Medicine, Hallym University Sacred Heart Hospital, Hallym University Medical School, Anyang, Republic of Korea

**Keywords:** Outcomes research, Chronic obstructive pulmonary disease

## Abstract

Acute exacerbation (AE) of chronic obstructive pulmonary disease (COPD) compromises health status; it increases disease progression and the risk of future exacerbations. We aimed to develop a model to predict COPD exacerbation. We merged the Korean COPD subgroup study (KOCOSS) dataset with nationwide medical claims data, information regarding weather, air pollution, and epidemic respiratory virus data. The Korean National Health and Nutrition Examination Survey (KNHANES) dataset was used for validation. Several machine learning methods were employed to increase the predictive power. The development dataset consisted of 590 COPD patients enrolled in the KOCOSS cohort; these were randomly divided into training and internal validation subsets on the basis of the individual claims data. We selected demographic and spirometry data, medications for COPD and hospital visit for AE, air pollution data and meteorological data, and influenza virus data as contributing factors for the final model. Six machine learning and logistic regression tools were used to evaluate the performance of the model. A light gradient boosted machine (LGBM) afforded the best predictive power with an area under the curve (AUC) of 0.935 and an F1 score of 0.653. Similar favorable predictive performance was observed for the 2151 individuals in the external validation dataset. Daily prediction of the COPD exacerbation risk may help patients to rapidly assess their risk of exacerbation and will guide them to take appropriate intervention in advance. This might lead to reduction of the personal and socioeconomic burdens associated with exacerbation.

## Introduction

Chronic obstructive pulmonary disease (COPD) is one of the leading causes of death worldwide (7.5–10%)^1,2^. In South Korea, COPD affects 13.6% and 30.5% of adults aged > 40 and > 65 years, respectively^3^; these values are higher than in other regions. COPD is a chronic inflammatory airway disease characterized by fixed airflow limitation and chronic respiratory symptoms (e.g., cough, sputum, and progressive dyspnea). Acute exacerbation can occur during the natural course of disease, and increasing disease progression. Acute exacerbation is an acute deterioration of health status, which leads patients to visit clinics earlier than scheduled and may be hospitalized. Exacerbation degrades the quality of life, decreases lung function, increases the risks of future exacerbation and mortality, and imposes socioeconomic burdens in terms of medical expenses and resources^4–8^. Severe exacerbations requiring hospitalization consume 70% of COPD-related healthcare expenditures. A claims-based analysis of 37,089 COPD patients in the U.S.A. found that the healthcare costs ranged from $2,003 to $43,461 per patient; they were substantially higher in patients who visited emergency departments and were hospitalized, particularly in intensive care units (ICUs)^9,10^. Although pharmacotherapies and interventional approaches improve disease control and outcomes, many patients continue to experience exacerbations.

If an acute exacerbation could be predicted and preparations could be made in advance, the individual and social burdens, as well as adverse outcomes, would be reduced. Several prediction models have been proposed for COPD exacerbation. In many cases, there models have not reached a sufficient level of high-quality statistical approach^11^. Additionally, there has been interest in single biomarkers for exacerbation prediction, but the significance of excluding clinical parameters has not been established^12^. Exacerbations are known to be triggered by a variety of factors including clinical and environmental variables such as respiratory viruses, pollution, and comorbidities^13–16^. In our previous study, we integrated individual medical information, weather and air pollution data, and the detection rates of respiratory viruses that affect acute COPD exacerbations^17^. Recently, a model predicting exacerbation on an annual basis has been developed^18^, but since exacerbation events do not occur at regular intervals and are influenced by changing environmental factors, it is essential to consider these factors.

Here, we developed prediction model for subsequent exacerbation event by integrating several information regarding the risk of exacerbation, then sequentially performed externally validation to identify its predictive power and adequacy.

## Methods

### Data source

We used six sources of data: the Korean COPD subgroup study (KOCOSS) cohort, the Korean Health Insurance Review and Assessment Service (HIRA), weather data, air pollution data collected by Air Korea^19^, trends in virus detection nationwide from the Korean Centers for Disease Control, and the Korean National Health and Nutrition Examination Survey (KNHANES) database.

#### KOCOSS cohort data

We used data from the KOCOSS cohort (registered on ClinicalTrials.gov with identifier CT02800499), an ongoing, prospective observational cohort of COPD, which has been recruiting patients from 48 referral hospitals in Korea since 2012.

Patients with COPD in the KOCOSS cohort were eligible if they were aged ≥ 40 years and exhibited a fixed airflow obstruction, as defined by a post-bronchodilator forced expiratory volume in 1 s (FEV_1_)/forced vital capacity (FVC) ratio < 0.7; detailed information concerning the KOCOSS cohort has been published^20^. The KOCOSS database contains information regarding lung function, smoking history, and disease-specific quality of life measured using the COPD assessment test (CAT).

#### HIRA data

HIRA receives medical claims data from all hospitals in South Korea, evaluates the appropriateness of the claims, and approves reimbursements from the National Health Insurance. All healthcare services rendered under national health insurance are claimed for reimbursement on a weekly or monthly basis, and are reported to HIRA and stored in HIRA’s in-house data warehouse. We collected information regarding age, sex, medications, comorbid conditions [classified using the International Classification of Disease, Tenth Revision (ICD-10) codes], and exacerbations.

#### Definition of exacerbation in HIRA database

Moderate exacerbation was defined by an outpatient visit with an COPD ICD-10 code (J43.x-44.x, except J430) with prescription of systemic steroids with or without antibiotics. Severe exacerbation was defined by a visit to an emergency room and/or hospital admission with a COPD (J43.x–J44.x, except for J43.0) or COPD-related diseases such as pneumonia (J12.x–J17.x), pulmonary thromboembolism (I26, I26.0 and I26.9), dyspnea (R06.0), or acute respiratory distress syndrome (J80) with prescriptions of steroid and/or antibiotics. This method has been utilized in many previous our studies^21–25^. COPD ICD-10 code, combined with the prescription of systemic steroids with or without antibiotics. All exacerbation was defined as an event for either moderate or severe exacerbation.

#### Meteorological and air pollution data

The Korean Ministry of the Environment provides daily local meteorological weather data including the levels of particulate matter (PM) of aerodynamic diameter < 10 μg/m^3^, minimum ambient temperatures (°C), and precipitation at local weather stations. These data are publicly available on the Ministry website. Due to the single, government-established health insurance system and the assignment of national identification (ID) number at birth, assessment results can be produced linking the ID number with healthcare data under the national insurance system. It enables us to match all weather information to patient addresses at the provincial level. Both meteorological and air pollution data were expressed as the number of days prior to the date of acute exacerbation.

#### Data on epidemic viruses

The Korean Centers for Disease Control and Prevention Agency (KDCA) has been conducting national surveillance for respiratory virus through the Korea Influenza and Respiratory Viruses Surveillance System (KINRRESS) since 2000. This system comprises two surveillance components: specimen-based and clinical surveillance systems. The former collects data from patients who visited outpatient clinics with acute respiratory illness at 52 designated sentinel sites. The latter gathers data from patients with severe disease who are admitted to hospitals. This nationwide surveillance system reports the weekly number of detected cases and positive rates of eight respiratory viruses including influenza virus, adenovirus, parainfluenza virus, respiratory syncytial virus, human coronavirus, human rhinovirus, human bocavirus, and human enterovirus using the multiplex reverse transcription-polymerase chain reaction (RT-PCR).These data are accessible to everyone on the KDCA website (http://www.kdca.go.kr/npt/).

### Model development and validation

All moderate to severe exacerbation were used as outcome variable, and the number of claims were used as individual event to analyze exacerbations. Based on each number of claims, participants of KOCOSS cohort were divided into training and internal validation dataset. We chose demographic data including age and sex, spirometry data, COPD related medications including inhaler treatment and systemic steroids and hospital visit for AE, air pollution data and meteorological data, and influenza virus data as relevant risk factors for the final model for prediction of moderate and/or severe exacerbation. Then, six machine learning and logistic regression tools were used to evaluate the performance of the model. Formula of prediction equation generated by GEE analysis was detailed in the supplementary methods.

The model will be reliable only if validated using data from other dataset. We validated our model using the KNHANES cohort; KNHANES conducts an annual, nationally representative study regarding the health and nutritional status of the South Korean population. We used data concerning 2151 individuals with FEV_1_/FVC ratios < 0.7; we employed only data from 2008 to 2012 (the years for which virus data were available). The KNHANES database does not include CAT scores; we calculated the scores using the EQ-5D values and patient ages^26^.

### Statistical analysis

The parameters used to develop the final predictive model are listed in Table 1. Seven statistical methods were used: random forest, extreme gradient boosting (XGBoost), light gradient boosted machine (LGBM), mixed-effect random forest (MERF), support vector machine (SVM), logistic regression, and generalized estimating equation (GEE). All models were taught using data resampled via adaptive synthetic sampling; this solved the binary class imbalance problem associated with acute exacerbation. We evaluated model performance in terms of accuracy and the area under the curve (AUC) of the receiving operating characteristic curve (see the statistical analysis of supplementary methods). Moreover, F1 score, sensitivity, positive predictive value, specificity and negative predictive value were also used as indicators for the possibility of low predictive power of model with very few events of interest and unbalanced dataset. Additional details are available in the Additional file: Supplementary Methods and Materials.

**Table 1:** Variables used in the prediction model.

Baseline characteristics
Age, sex, height, weight, FEV_1_% of predicted value, CAT score, smoking status, pack-year, address in provinces
Drug information: number of drug prescription
LABA and/or LAMA, ICS, ICS/LABA, SAMA or SABA, systemic corticosteroids, LTRA, theophylline
Frequency of hospital visits due to COPD
Frequency of hospital visits for diseases other than COPD
Ischemic heart disease, lung cancer, osteoporosis, depression, arthritis, diabetes mellitus, gastroesophageal reflux disease, pneumothorax, congestive heart failure, hypertension, anemia, metabolic syndrome
Information of hospital visit during last year
Date since last COPD related hospital visit, whether referral hospital visit in last visit
Frequency of acute exacerbation
Numbers of visiting referral hospital for exacerbation within a year
Meteorological and air pollution data
Maximum and average concentration of PM_10_, NO_2_, PM_2.5_ within a week and 42 days
Mean temperature, Maximum diurnal temperature, average humidity within a week
Epidemic respiratory virus
Detection rate of influenza virus detection

*CAT* COPD assessment test, *COPD* chronic obstructive pulmonary disease, *FEV*_*1*_ forced expiratory volume in 1 s, *ICS* inhaled corticosteroid, *LABA* long-acting beta2 receptor agonist, *LAMA* long-acting muscarinic receptor agonist, *LTRA* leukotriene receptor antagonists, *NO*_*2*_ nitrogen dioxide, *PM* particulate matter, *SABA* short-acting beta2 receptor agonist, *SAMA* short-acting muscarinic receptor agonist.

We divided the 590 KOCOSS patients into training and internal validation test sets by splitting 18,945 individual claims, rather than splitting them by each patient's identification number. The final model selection was based on an internal validation set that closely resembled the modeling training dataset. Additionally, the performance evaluation of the final model was conducted on an external validation dataset, the KNHANES. The KNHANES database was then used to explore predictive performance. We conducted an analysis of the performance of each prediction model through 50 replicates of bootstrapping for each, presenting results for each metric in terms of mean value and the lower and upper value of the 95% confidence interval (CI). All models were implemented in Python 3.7 and run in the Windows 10 environment.

### Ethics approval and consent to participate

All hospitals involved in the KOCOSS cohort obtained approval from their respective institutional review board committees and informed consent from their patients. The study protocol was approved by the Institutional Review Board of the KONKUK University Medical Center (IRB No. KHH1010338). This study used data from KNHANES as validation cohort and KNHANES was approved by the Institutional Review Board of the Korea Centers for Disease Control (IRB No. 1401–047-547). The patients/participants provided their written informed consent to participate in this study.

## Results

### Characteristics of participants

**Figure 1:**
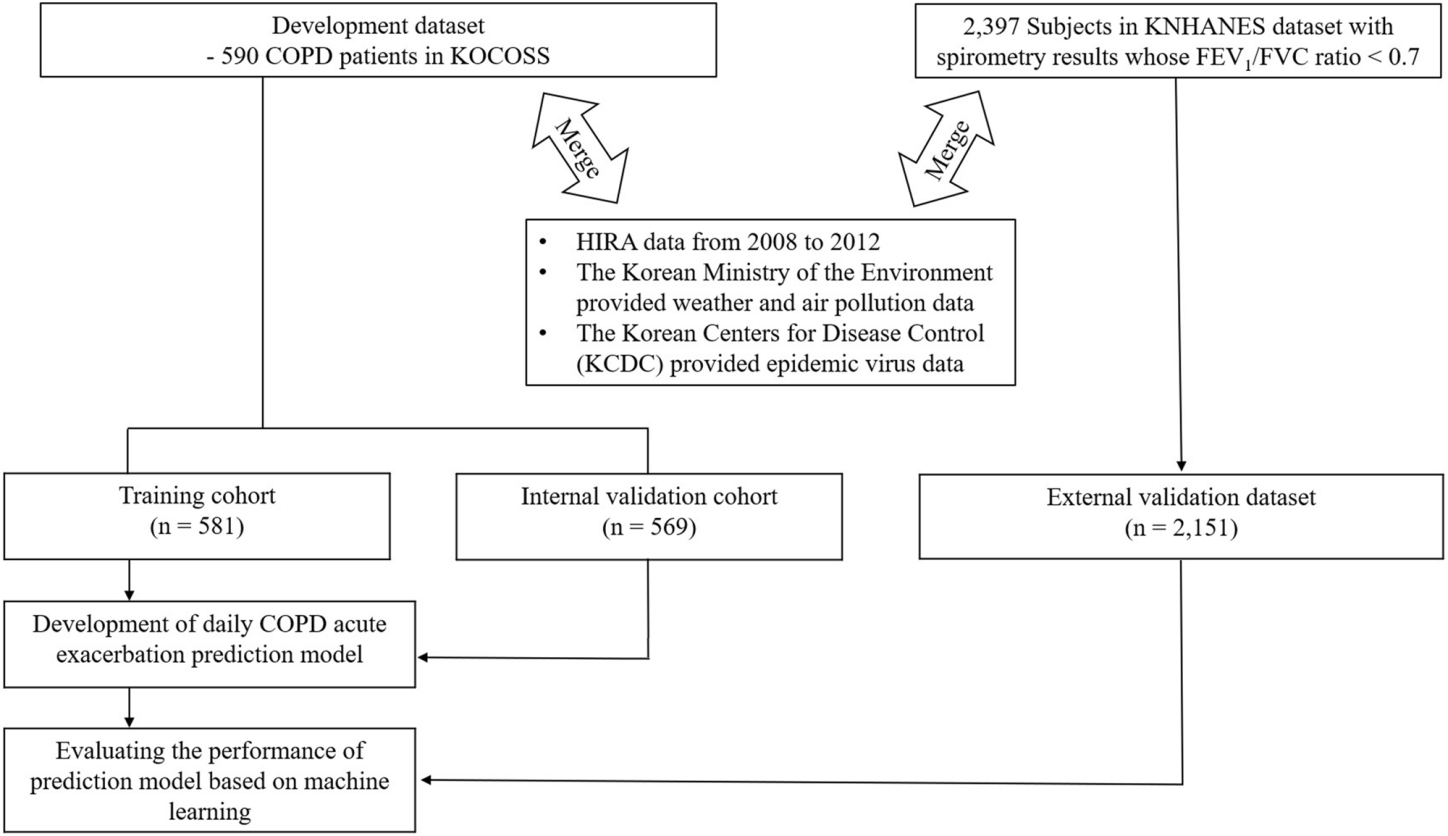
Scheme of study design. *COPD* chronic obstructive pulmonary disease, *FEV*_*1*_ forced expiratory volume in 1 s, *FVC* forced vital capacity, *HIRA* the Korean Health Insurance Review and Assessment Service, *KNHANES* Korean National Health and Nutrition Examination Survey, *KOCOSS* Korean COPD Subgroup Study.

The 590 COPD patients in the KOCOSS cohort were randomly divided into a training subset (n = 581) and an internal validation subset (n = 569) based on their number of claims (Fig. 1). Both sets were similar in terms of sociodemographic features, severity of airflow limitation, and disease-related quality of life (Table 2). Over 90% of all patients were men; the mean age was approximately 65 years, and over 80% of all patients were current or ex-smokers. The mean FEV_1_ was approximately 52% of the predicted value in both groups. The mean numbers of all exacerbations were 1.05 ± 2.07/year in the training subset and 2.53 ± 2.45/year in the validation subset. The mean numbers of severe exacerbations were 0.08 ± 0.24/year and 0.17 ± 0.43/year, respectively.

**Table 2:** Comparison of baseline characteristics between training, internal validation and external validation dataset.

Characteristics	KOCOSS		KNHANES
	Training cohort (n = 581)	Internal validation cohort (N = 569)	External validation cohort (N = 2151)
Age, year	65.6 ± 7.4	64.8 ± 7.4	63.9 ± 11.6
Male	524 (90.2)	513 (90.2)**	1,521 (70.7)**
Height, cm	163.8 ± 7.3	163.7 ± 7.3*	162.9 ± 8.7*
Weight, kg	61.2 ± 10.6	61.2 ± 10.5*	62.7 ± 10.3*
Smoking status			
Current	145 (25.0)	139 (24.4)**	645 (30.0)**
Ex	343 (59.0)	339 (59.6)**	377 (17.5)**
Non	6 (1.0)	6 (1.1)**	665 (30.9)**
Smoking, pack-year	37.9 ± 27.4	37.7 ± 27.2**	14.7 ± 21.3**
Lung function			
FEV_1_, % of predicted value	51.9 ± 16.1	52.0 ± 16.5**	78.6 ± 13.7**
FEV_1_/FVC ratio	61.4 ± 10.6	61.3 ± 10.6**	64.0 ± 6.0**
CAT score	16.2 ± 7.1	16.2 ± 7.1**	10.3 ± 13.6**

Data are presented as the mean ± standard deviation or number (%).

*CAT* COPD assessment test, *COPD* chronic obstructive pulmonary disease, *FEV*_*1*_ forced expiratory volume in 1 s, *FVC* forced vital capacity, *KNHANES* the Korean National Health and Nutrition Examination Survey, *KOCOSS* the Korean COPD subgroup study.

* < 0.05, ** < 0.001; statistical difference represents the comparative value between internal and external validation sets.

The external validation cohort included 2151 individuals in the KNHANES dataset with FEV_1_/FVC ratios < 0.7, 70.7% of whom were men; the mean age was 64 years. Compared with the KOCOSS cohort, there were more non-smokers (30.9% vs. 1%), the FEV_1_ was higher (78.5% vs. 52.1% of the predicted value), and the CAT score was lower (10.3 vs. 16.1) in the KNHANES cohort (Table 2).

### Development of an exacerbation prediction model

A scheme of the study design is shown in Fig. 1. Environmental data including information of weather, air pollution and epidemic respiratory virus, individual’s personal data from KOCOSS cohort and data of HIRA dataset of each participant. Each item was assumed affecting AE and represented by odds ratio (OR). The ORs for the personal factors including demographic data, comorbid conditions and hospital visits for COPD and/or AE were found Table S1 and ORs for the respiratory medications were presented in Table S2 in the additional file. Effects of air pollution, weather and virus trends on AE was presented in Table S3 in the additional file. Only significant variables with a p value of 0.05 or less in these univariable results were selected and the multivariable model was applied to confirm the ORs of each variable, and through this approach, final prediction model was generated (Table 3). We chose age, sex, smoking status, comorbidities, FEV_1_ value, disease specific medication usage and hospital visit, frequency of AE, air pollution data (e.g., NO_2_ and PM_2.5_), humidity and diurnal temperature data, and epidemic influenza virus data in final model.

**Table 3:** Factors associated with COPD AE estimated by a generalized estimation equation model through multivariate logistic regression analysis.

Variable	OR	p-value	95% CI
Male sex	0.756	0.030	0.587–0.973
Smoking status			
Never	0.215	0.059	0.043–1.059
Ex	1.018	0.885	0.796–1.302
Current	1.133	0.379	0.857–1.498
Post-BD FEV_1_ of % predicted value	0.995	0.051	0.989–1.000
Number of times ICS/LABA was taken in 365 days	1.033	0.011	1.007–1.058
Number of times LAMA was taken in 90 days	0.906	0.031	0.829–0.991
Number of times SABA was taken in 182 days	1.047	0.024	1.006–1.089
Number of times OCS was taken in 90 days	1.125	0.000	1.078–1.173
Number of hospital visits with anemia in 365 days	0.877	0.002	0.806–0.954
Number of hospital visits with COPD in 182 days	0.877	0.000	0.850–0.904
Cumulative numbers of AEs	1.032	0.019	1.005–1.059
Cumulative numbers of severe AEs	1.155	0.002	1.052–1.269
Number of AEs in 182 days	1.256	0.000	1.165–1.355
Number of AEs in 90 days	1.319	0.000	1.178–1.477
Average NO_2_ value in 42 days	0.000	0.000	0.000–0.000
Average PM_2.5_ value in 42 days	1.015	0.009	1.004–1.026
Average humidity for a week	0.992	0.008	0.986–0.998

*AE* acute exacerbation, *CI* confidence interval, *COPD* chronic obstructive pulmonary disease, *FEV*_*1*_ forced expiratory volume in 1 s, *FVC* forced vital capacity, *ICS* inhaled corticosteroid, *IFV* influenza virus, *LABA*,long acting beta2 receptor agonist, *LAMA* long-acting muscarinic receptor antagonist, *NO*_*2*_ nitrogen dioxide, *OCS* oral corticosteroids, *OR* *PM*_*2.5*_ particulate matter (PM) with aerodynamic diameters ≤ 2.5 μm, *SABA* long acting beta2 receptor agonist.

**Figure 2:**
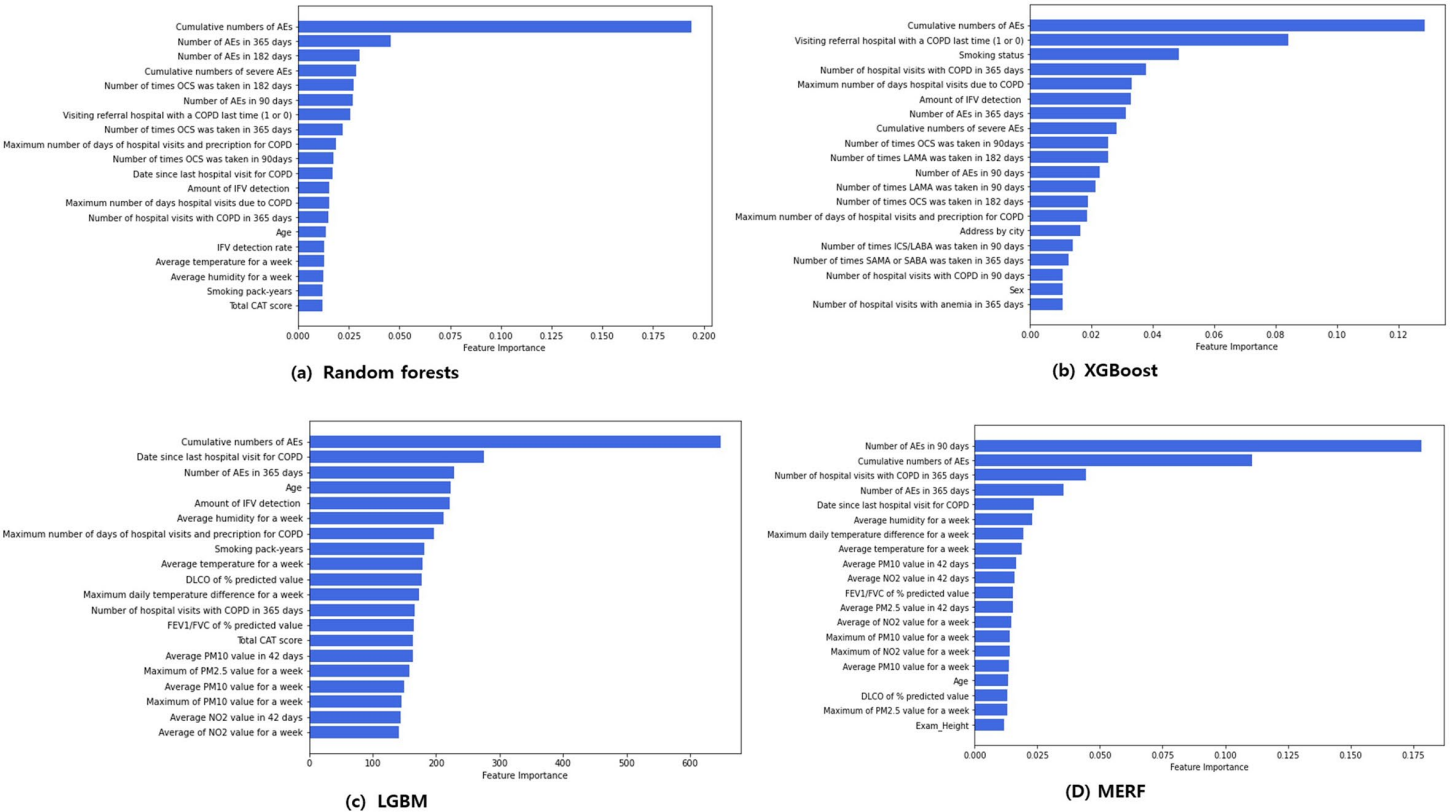
Impurity based feature importance of tree based machine learning methods. *AE* acute exacerbation, *CAT* chronic obstructive pulmonary disease (COPD) assessment test, *DLCO* diffusing capacity for carbon monoxide, *FEV*_*1*_ forced expiratory volume in 1 s, *FVC* forced vital capacity, *ICS* inhaled corticosteroid, *IFV* influenza virus, *LABA* long-acting beta2 receptor agonist, *LAMA* long-acting muscarinic receptor antagonist, *NO*_*2*_ nitrogen dioxide, *OCS* oral corticosteroids, *PM* particulate matter, *SABA* short-acting beta2 receptor agonist.

In Fig. 2, we showed the feature importance for the (a) random forest, (b) XGBoost, (c) LGBM, and (d) MERF model, which was made by impurity-based method for feature selection that best splits the dataset. Features that tend to split dataset more precisely will result in a large importance value in that order. Past exacerbation events were most important feature in all machine learning model.

### Internal validation of the predictive model

We fitted seven models and prediction performance in internal validation cohort is presented in Table S4 in the additional file. The cutoffs of the perceived exacerbation risks were values that maximized model performance. An SVM classifies data by finding the optimal hyperplane that separates all data in a binary manner (i.e., in one group or the other); no cutoff is given. The performance metrics were the AUC, accuracy, F1 score, recall, precision, specificity and negative predictive value. All bootstrapped data of each metric were presented in terms of mean value and upper and lower value of the 95% CI. The LGBM model was optimal in terms of AUC, accuracy, F1 score and sensitivity, specificity and negative predictive value. The random Forest, XGBoost, and MERF models also excellently predicted the risk of future exacerbation.

### External validation of the predictive model

The results of external validation are shown in Table 4. The random forest, XGBoost, LGBM, and MERF models exhibited similarly high predictive capacities, according to all parameters. All four models had AUCs and F1 scores > 0.7; furthermore, the random forest and MERF models yielded F1 scores > 0.8.

**Table 4:** Prediction performance in validation cohort (KNHANES).

	Mean	95% CI lower limit	95% CI upper limit
Random forest			
AUC	0.737	0.725	0.750
Accuracy	0.766	0.759	0.775
F1 score	0.841	0.837	0.846
Sensitivity (Recall)	0.992	0.989	0.994
PPV (Precision)	0.730	0.725	0.737
Specificity	0.390	0.374	0.412
NPV	0.965	0.957	0.974
Cut-off value	0.3		
XGBoost			
AUC	0.734	0.721	0.753
Accuracy	0.678	0.669	0.687
F1 score	0.735	0.728	0.742
Sensitivity (Recall)	0.717	0.706	0.726
PPV (Precision)	0.755	0.747	0.765
Specificity	0.613	0.598	0.634
NPV	0.566	0.555	0.576
Cut-off value	0.3		
LGBM			
AUC	0.720	0.708	0.734
Accuracy	0.713	0.704	0.724
F1 score	0.784	0.777	0.792
Sensitivity (Recall)	0.831	0.822	0.843
PPV (Precision)	0.741	0.733	0.750
Specificity	0.518	0.499	0.537
NPV	0.649	0.634	0.668
Cut-off value	0.211		
MERF			
AUC	0.721	0.711	0.733
Accuracy	0.769	0.765	0.773
F1 score	0.844	0.841	0.846
Sensitivity (Recall)	1.000	1.000	1.000
PPV (Precision)	0.730	0.726	0.734
Specificity	0.386	0.373	0.397
NPV	1.000	1.000	1.000
Cut-off value	0.22		
Logistic			
AUC	0.537	0.521	0.548
Accuracy	0.441	0.436	0.447
F1 score	0.297	0.286	0.311
Sensitivity (Recall)	0.189	0.180	0.199
PPV (Precision)	0.692	0.674	0.711
Specificity	0.860	0.849	0.874
NPV	0.390	0.386	0.393
Cut-off value	0.5		
SVM			
AUC	0.541	0.535	0.551
Accuracy	0.476	0.471	0.485
F1 score	0.401	0.390	0.414
Sensitivity (Recall)	0.281	0.270	0.292
PPV (Precision)	0.702	0.691	0.721
Specificity	0.802	0.790	0.814
NPV	0.401	0.398	0.407
Cut-off value	–		
GEE			
AUC	0.540	0.528	0.550
Accuracy	0.465	0.455	0.472
F1 score	0.398	0.385	0.408
Sensitivity (Recall)	0.283	0.271	0.292
PPV (Precision)	0.668	0.652	0.683
Specificity	0.766	0.754	0.780
NPV	0.391	0.386	0.397
Cut-off value	0.2		

*AUC* area under the curve, CI confidence interval, *GEE* generalized estimating equation, *KOCOSS* the Korean COPD subgroup study, *LGBM* light gradient boosted machine, *MERF* mixed effect random forest, *NPV* negative predicted value, *PPV* positive predictive value, *SVM* support vector machine, *XGBoost* extreme gradient boosting.

### Examples of application of the prediction model

Various personal and environmental factors can be fed into machine learning models to predict daily exacerbation risk. To demonstrate the performance of our predictive model, we tested two hypothetical scenarios. We presented two representative cases in which the risk of exacerbation was assessed differently at each independent date (Fig. S1). Patient in case A has low personal risk factors and the probability of exacerbation in a favorable environment is 11%. While, B has high risk factors and the probability in unfavorable environment is 97%.

## Discussion

We developed a model for predicting COPD exacerbation on a daily basis by merging clinical cohort data, 5 years of follow-up claims data, and information regarding air pollution and respiratory virus infection rates. Those with spirometry confirmed airflow limitation in the nationally representative database that contains 5 years of data regarding claims and external factors were used as validation cohort. We found similar overall favorable predictive performance especially the random forest, XGBoost, LGBM, and MERF models exhibited good predictive power. Our personalized model to predict exacerbation was created using data from patients with symptomatic COPD who were treated and followed-up in referral hospitals, but also has high predictive ability in patients with less symptomatic, mild, and undiagnosed COPD. This suggestive the possibility of universal application of our tool.

In our previous study of 594 COPD patients in the KOCOSS cohort, the HIRA data from 2007 to 2012 were merged and the relationships of future exacerbation with various clinical and environmental factors were analyzed^17^. We showed female sex (adjusted odds ratio [aOR], 1.5596; 95% confidence interval [CI], 1.1742–2.0715), number of exacerbations during the previous year (aOR, 1.2745; 95% CI 1.2095–1.3430), COPD grade (aOR, 1.5693; 95% CI 1.1727–2.1001), influenza virus detection rate 2 weeks before exacerbation (aOR, 1.0075; 95% CI 1.0017–1.0133), the lowest temperature in the 5 days before exacerbation (aOR, 0.9572; 95% CI 0.9219–0.9938), and the lowest temperature 1 day before exacerbation (aOR, 0.9587; 95% CI 0.9196–0.9994) were significantly associated with exacerbation. Based on the previous study, this study represented an effort to assess the risk of exacerbation prediction by incorporating not only demographic and clinical variables commonly used in previous researches but also environmental factors known to influence exacerbations. This aspect constitutes a significant strength of this study and we developed a predictive model of exacerbation and verified predictive performance through machine learning tools. Moreover, among various clinical and environmental factors, we identified the relative importance of each factor on exacerbation on each machine learning method. This is the first attempt and one of novel and interesting finding in this study.

To our knowledge, this is the first daily prediction model for exacerbation of COPD patients worldwide; our enhancement of predictive power via machine learning is novel. We used daily exacerbation data collected over several years; we also used daily air pollution, climate, and epidemic respiratory information. Korea’s unique health insurance system and readily accessible big data enable data collection. Few studies have sought to predict the risk of acute exacerbation, particularly on a daily basis, for several reasons. First, sufficient data have been lacking. We used information from the KOCOSS multicenter cohort; we validated the model using information from a nationwide health survey (KNHANES) and claims data. Therefore, our model is applicable to mild, asymptomatic, underdiagnosed, and symptomatic COPD patients. Importantly, it was possible to identify hospital visits caused by exacerbation. In 1998, South Korea implemented a National Health Insurance system that covers almost all of the population^27^. All healthcare institutions in South Korea claim medical expenses through HIRA, which evaluates the appropriateness of claims and approves National Health Insurance reimbursements. HIRA collects patient information provided by physicians; data concerning almost all COPD patients and their healthcare requirements can be found in the HIRA database. Because of this accurate database, it was possible to develop a predictive formula. The model includes severity of COPD itself, which is represented by lung function. The claims data lack pulmonary function test results. However, because we merged claims, cohort, and KNHANES data, we integrated lung function into our model. Second, because COPD is chronic in nature, most cohort data were derived during long-term follow-up, rendering it difficult to engage in statistical modeling because data independence may have been violated by the collection of repeated measurements. To overcome this correlation problem with respect to longitudinal data, the GEE and two machine learning methods (random forest and deep neural network) based on decision trees (in which independence is not assumed) were used to develop the predictive model. Third, event imbalance before and after resampling must be considered (see Table S5). The incidence of acute COPD exacerbation was low; only 3% of hospital visits by COPD patients were triggered by such exacerbation. Most COPD patients do not experience acute exacerbations on most days. The adaptive synthetic sampling method was applied to all machine learning models; this reduced the problems caused by event imbalances.

The airflow limitation group (FEV_1_/FVC ratios < 0.7) from the KNHANES dataset with an average FEV_1_ of 79% of predicted value was used as an external validation cohort. In general, approximately 75% of COPD patients remain undiagnosed because most of them are less symptomatic^28^. Likewise, only less than 5% of individuals are reported to visit and treated for COPD in South Korea^29^. Most of these less symptomatic patients that hardly voluntarily visit hospital, thus it is difficult to find these group of individuals. However, a general population-based studies showed that although these undiagnosed COPD patients are a- or less symptomatic, they are at higher risk of exacerbation and even mortality^30,31^. Our predictive model was derived from overt COPD cohort and validated in less symptomatic individuals from general population cohort. This suggests the generalizability of the model to patients with mild to severe COPD.

In this study, we created a predictive model for exacerbation that considered both external and clinical factors. The use of machine learning to improve the predictive power is both novel and encouraging. An exacerbation episode is not occurred with clear time interval with sharp start and end. But rather than it is a gradual process with crescendo and decrescendo process. Our prediction model could be applied regardless of any distinct time interval in predicting the risk of near future. If patients can easily assess their own daily risk of exacerbation, it may enable preventive interventions such as educating patients to wear masks or hand hygiene during respiratory virus outbreaks. Additionally, when there is an overall increase in exacerbation risk, patients could reschedule their outpatient visits, allowing for timely preventive interventions. However, our work had some limitations. First, because exacerbations were identified using claims data, over- or underestimation was possible. However, we combined the ICD-10 code with the prescribed drug; this may have rendered the data robust. Second, the PM level was based on the patient address in the HIRA database; this address was presumably not always the location where the exacerbation occurred. An association between PM level and COPD exacerbation has been found in many epidemiological studies. However, South Korea is a small country (total area 100,210 km^2^). Thus, the extent of air pollution may not greatly differ among regions. Third, we have taken into account weather variables prior to exacerbation, however, we were unable to precisely apply for the clear time lag. Fourth, high-quality data are available regarding weather conditions, PM levels, and epidemic respiratory virus status in Korea; it was thus possible to predict exacerbation considering all of these factors. However, it may be difficult to generalize the application of our predictive tool to other countries where such data are not readily available to the public. Thus, it seems necessary to develop an exacerbation model using universally available and applicable information. Fourth, we analyzed the influence of each drug type but did not incorporate levels of potency of each drug.

In conclusion, because COPD exacerbations negatively impact health and disease progression including further exacerbation, early detection of at-risk patients is helpful to response timely and appropriately. Exacerbation is triggered by combinations of various clinical and environmental factors. To our knowledge, we are the first group worldwide to develop a personalized, daily, predictive, COPD exacerbation model; we integrated various relevant internal and external factors including multicenter cohort and claims data, as well as big data concerning climate, air pollution, and epidemic respiratory virus status. We improved the model’s predictive power by using several machine learning methods. Our model will encourage patients to visit the hospital early when they aware an increased risk of exacerbation; timely and appropriate intervention will follow.

### Supplementary Information

Supplementary Figure S1.

Supplementary Information 1.

Supplementary Information 2.

## Data Availability

The datasets used for the current study are available from the corresponding author upon reasonable request. Also, publicly available datasets (KNHANES) can be found here: https://knhanes.kdca.go.kr/knhanes/main.do.

## Abbreviations

CAT: Chronic obstructive pulmonary disease assessment test
COPD: Chronic obstructive pulmonary disease
FEV_1_: Forced expiratory volume in 1 s
FVC: Forced vital capacity
GEE: Generalized estimating equation
KOCOSS: The Korean COPD subgroup study
KNHANES: The Korean National Health and Nutrition Examination Survey
LGBM: Light gradient boosted machine
PM: Particulate matter
MERF: Mixed-effect random forest
SVM: Support vector machine
XGBoost: Extreme gradient boosting
