# Supplementary figures and images for: Development of a daily predictive model for the exacerbation of chronic obstructive pulmonary disease
Source: Sci Rep. 2023 Oct 31;13:18669. doi: 10.1038/s41598-023-45835-4 (PMC10618439; doi:10.1038/s41598-023-45835-4)

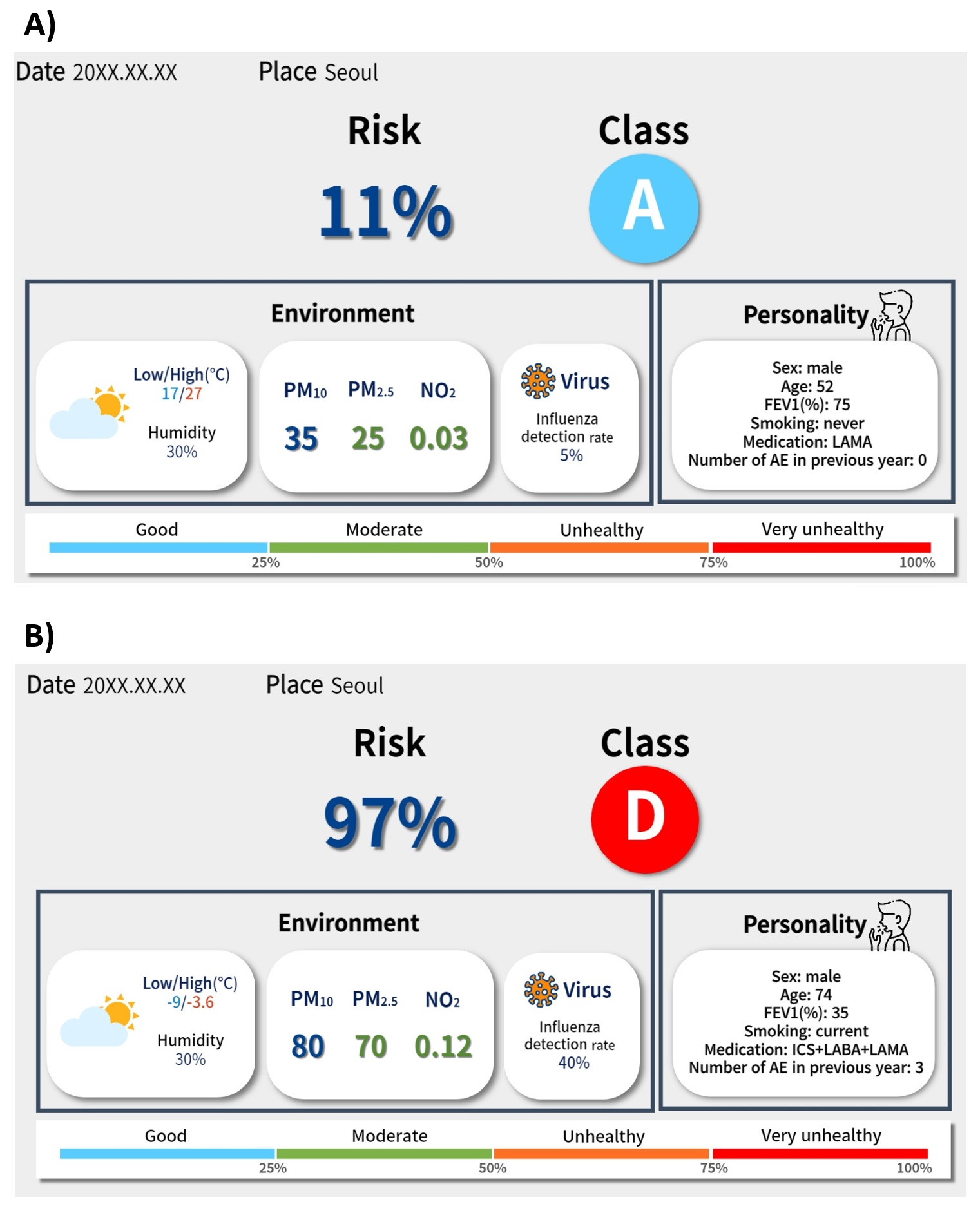

Supplement: Supplementary file 1 — Supplementary Figure S1. [file 41598_2023_45835_MOESM1_ESM.tiff]
