# Supplementary material for: Development of a daily predictive model for the exacerbation of chronic obstructive pulmonary disease
Source: Sci Rep. 2023 Oct 31;13:18669. doi: 10.1038/s41598-023-45835-4 (PMC10618439; doi:10.1038/s41598-023-45835-4)
Supplement: Supplementary file 2 — Supplementary Information 1. [file 41598_2023_45835_MOESM2_ESM.docx]

**"Online Supplementary Materials"**

**Development of a daily predictive model for the exacerbation of chronic obstructive pulmonary disease**

**Python command code for each analysis method used in machine learning**

**# Import the library**

from sklearn.model_selection import RepeatedStratifiedKFold

from skopt import BayesSearchCV

from merf.merf import MERF

from imblearn.over_sampling import ADASYN

from sklearn.linear_model import LogisticRegression

from sklearn import svm

import statsmodels.formula.api as smf

from sklearn.metrics import accuracy_score, f1_score, recall_score, precision_score, classification_report, confusion_matrix, roc_auc_score

from sklearn.model_selection import train_test_split

**Resampling & Data-preprocessing**

# Train/Test

X = df_model.drop(['acute','severe'], axis=1)

y = df_model['acute']

X_train, X_test, y_train, y_test = train_test_split(X, y, test_size=0.33, random_state=42)

# Resampling

ada = ADASYN(random_state=915)

X_a, y_a = ada.fit_resample(X_train, y_train)

# Pre-processing

X_train_gee = X_train.copy()

X_test_gee = X_test.copy()

X_train = X_train.drop('hpin',axis=1)

X_test = X_test.drop('hpin',axis=1)

gee_train = pd.merge(X_train_gee,y_train,how='left',left_index=True,right_index=True)

gee_test = pd.merge(X_test_gee,y_test,how='left',left_index=True,right_index=True)

gee_valid = pd.merge(X_valid_gee,y_valid,how='left',left_index=True,right_index=True)

X_train_merf = X_train.copy() ; X_train_merf['z']=1

X_test_merf = X_test.copy() ; X_test_merf['z']=1

**Evaluation function**

def BinaryClassificationReport(model, testx, testy, thresh):

# Prediction

pred_proba = model.predict_proba(testx)

pred = (pred_proba[:,1] > thresh).astype('float')

# Report

print(f"""

Classification Report for model : {model}

****result indexes****

accuracy : {accuracy_score(testy, pred) :.4f}

f1 score : {f1_score(testy, pred) :.4f}

Recall score : {recall_score(testy, pred) :.4f}

Precision score : {precision_score(testy, pred) :.4f}

AUC(AUROC) : {roc_auc_score(testy, pred_proba[:,1]) :.4f}

***Confusion matrix***

{confusion_matrix(testy, pred)}

*Classification report*

{classification.classification_report(testy, pred, n=500)}

""")

def BinaryClassificationReportMerf(model, testx, testy, thresh):

#predications

pred_proba = model.predict(testx, testx[['z']], testx['hpin'])

res = pd.DataFrame(pred_proba)

res.loc[res[0]>thresh, 'res'] = 1

res.fillna(0, inplace=True)

pred = res['res']

#reports

print(f"""

Classification Report for model : {model}

****result indexes****

accuracy : {accuracy_score(testy, pred) :.4f}

f1 score : {f1_score(testy, pred) :.4f}

Recall score : {recall_score(testy, pred) :.4f}

Precision score : {precision_score(testy, pred) :.4f}

AUC(AUROC) : {roc_auc_score(testy, pred_proba) :.4f}

***Confusion matrix***

{confusion_matrix(testy, pred)}

*Classification report*

{classification_report(testy, pred, digits=4)}

""")

def SVMres(model, testx, testy):

#predications

pred = model.predict(testx)

#reports

print(f"""

Classification Report for model : {model}

****result indexes****

accuracy : {accuracy_score(testy, pred) :.4f}

f1 score : {f1_score(testy, pred) :.4f}

Recall score : {recall_score(testy, pred) :.4f}

Precision score : {precision_score(testy, pred) :.4f}

AUC(AUROC) : {roc_auc_score(testy, pred) :.4f}

***Confusion matrix***

{confusion_matrix(testy, pred)}

*Classification report*

{classification_report(testy, pred, digits=4)}

""")

def GEEBinaryClassificationReport(model, testx, testy, thresh):

#predications

pred_proba = model.fit()._results.predict(exog = testx , linear = False )

pred = (pred_proba > thresh).astype('float')

#reports

print(f"""

Classification Report for model : {model}

****result indexes****

accuracy : {accuracy_score(testy, pred) :.4f}

f1 score : {f1_score(testy, pred) :.4f}

Recall score : {recall_score(testy, pred) :.4f}

Precision score : {precision_score(testy, pred) :.4f}

AUC(AUROC) : {roc_auc_score(testy, pred_proba) :.4f}

***Confusion matrix***

{confusion_matrix(testy, pred)}

*Classification report*

{classification_report(testy, pred , digits=4)}

""")

**Parameter Optimization**

def val_experiment (search, X_train, y_train, X_test, y_test, X_valid, y_valid, thresh, folds=10) :

search.fit(X_train,y_train)

# optimal parameter

result = search.best_estimator_

result.fit(X_train,y_train)

# FI with test set

plt.show(fimpt(X_test,result))

#evaluation

BinaryClassificationReport( result,X_test,y_test, thresh)

BinaryClassificationReport( result,X_valid, y_valid, thresh)

return(result)

# randomforest

rf_params = [{'n_estimators': [100,150,200],

'max_depth': [None, 5,10,15],

'min_samples_split': [ 5, 10],

'min_samples_leaf' : [4,8]}]

rf_search = BayesSearchCV(estimator=RandomForestClassifier(), search_spaces=rf_params,

random_state = 42, n_jobs=-1, cv=5, verbose=0)

rf_result = val_experiment (search=rf_search, X_train =X_train, y_train=y_train, X_test=X_test, y_test=y_test, X_valid=X_valid, y_valid=y_valid, thresh=0.3, folds=5)

rf_ada_result = val_experiment (search=rf_search, X_train= X_a, y_train=y_a, X_test=X_test, y_test=y_test, X_valid=X_valid, y_valid=y_valid, thresh=0.4, folds=5)

# xgboost

xgb_param = [{'n_estimators' : [ 100, 300, 500],

'reg_lambda' : [0, 0.25, 0.5, 1, 1.5],

'reg_alpha' : [0, 0.25, 0.5, 1.5] }]

xgb_search = BayesSearchCV(estimator=XGBClassifier(), search_spaces=xgb_param,

random_state = 42, n_jobs=-1, cv=5, verbose=0)

xgb_result = val_experiment (search=xgb_search, X_train =X_train, y_train=y_train, X_test=X_test, y_test=y_test, X_valid=X_valid, y_valid=y_valid, thresh=0.21, folds=5)

xgb_ada_result = val_experiment (search=xgb_search, X_train=X_a, y_train=y_a, X_test=X_test, y_test=y_test, X_valid=X_valid, y_valid=y_valid, thresh=0.21, folds=5)

# LGBM

lgbm_param = [{ 'n_estimators' :[100, 200, 400 ],

'max_depth': [2, 3, 5, 8, 10 ],

'subsample' : [0.5, 0.7, 1] }]

lgbm_search = BayesSearchCV(estimator=LGBMClassifier(), search_spaces=lgbm_param,

random_state = 42, n_jobs=-1, cv=5, verbose=0)

lgbm_result = val_experiment (search=lgbm_search, X_train =X_train, y_train=y_train, X_test=X_test, y_test=y_test, X_valid=X_valid, y_valid=y_valid, thresh=0.19, folds=5)

lgbm_ada_result = val_experiment (search=lgbm_search, X_train=X_a, y_train=y_a, X_test=X_test, y_test=y_test, X_valid=X_valid, y_valid=y_valid, thresh=0.211, folds=5)

**"""**

**modeling & evaluation**

**"""**

# Randomforest

rf_ada = RandomForestClassifier(random_state=42, n_estimators=150, n_jobs=-1)

rf_ada.fit(X_a, y_a)

BinaryClassificationReport(rf_ada,X_test,y_test, 0.3)

BinaryClassificationReport(rf_ada,X_valid,y_valid, 0.3)

# XGBoost

xgb_ada = XGBClassifier(n_estimators=100, random_state=42,

base_score=0.5, booster='gbtree', colsample_bylevel=1,

colsample_bynode=1, colsample_bytree=1, gamma=0, gpu_id=-1,

importance_type='gain', interaction_constraints='',

learning_rate=0.300000012, max_delta_step=0, max_depth=6,

min_child_weight=1, missing=np.nan, monotone_constraints='()',

n_jobs=0, num_parallel_tree=1,

reg_alpha=0, reg_lambda=1, scale_pos_weight=1, subsample=1,

tree_method='exact', validate_parameters=1, verbosity=None)

xgb_ada.fit(X_a, y_a)

BinaryClassificationReport(xgb_ada,X_test,y_test, 0.3)

BinaryClassificationReport(xgb_ada,X_valid,y_valid, 0.3)

# LGBM

lgb_ada = LGBMClassifier(n_estimators=100, num_leaves=80, random_state=42)

lgb_ada.fit(X_a,y_a)

BinaryClassificationReport(lgb_ada,X_test,y_test, 0.211)

BinaryClassificationReport(lgb_ada,X_valid,y_valid, 0.211)

# MERF

max_iter = 5

merf_base = MERF(max_iterations=max_iter)

merf_base.fit(X_train_merf, X_train_merf[['z']], X_train_merf['hpin'], y_train)

BinaryClassificationReportMerf(merf_base, X_test_merf, y_test, 0.22)

BinaryClassificationReportMerf(merf_base, X_valid_merf, y_valid, 0.22)

#logistic

logit = LogisticRegression(solver='liblinear')

logit.fit(X_a, y_a)

BinaryClassificationReport(logit,X_test,y_test, 0.5)

BinaryClassificationReport(logit,X_valid,y_valid, 0.5)

#SVM

svm_res_ada = svm.SVC(kernel='rbf',probability=True)

svm_res_ada.fit(X_a, y_a)

SVMres(svm_res_ada,X_test,y_test)

SVMres(svm_res_ada,X_valid,y_valid)

SVMres(svm_res_ada,X_valid,y_valid)

# GEE

def MultipleGee(No,formula, df) :

fam = sm.families.Binomial()

ind =sm.cov_struct.Independence()

mod = smf.gee(formula, 'hpin', data=df, cov_struct=ind, family=fam)

tb= pd.DataFrame(mod.fit().summary().tables[1], columns=None)[1:]

tb.columns = ["variable", "coef","std err", "z" , "P>|z|" , "LB", "UB" ]

names = "/Tables/GEEmultiEst" + No + "_indep.csv"

tb.to_csv(names, index=True )

odds = pd.DataFrame(np.exp(mod.fit().params), columns= ['OR'])

odds['p-value']= mod.fit().pvalues

odds[['2.5%', '97.5%']] = np.exp(mod.fit().conf_int())

names = "/Tables/GEEmultiodds" + No + "_indep.csv"

odds.to_csv(names, index=True )

return (mod)

var = gee_train.columns.to_list()

del var[0], var[-1]

formula = '+'.join(var)

gee = smf.gee('acute ~' + formula, "hpin", gee_train)

res = gee.fit()

print(res.summary())

for11= 'acute~ C(sex) + FEV1_pcFore +C(His_SmokeNow) + PreCnt365_ICSLABA + PreCnt90_LAMA + PreCnt182_SABA + PreCnt90_OCS + PreCnt365_C11 + PreCnt182_copd + cum_acute + cum_severe + PreCnt182_acute + PreCnt90_acute + NO2_mean42 + pm25_mean42 + hum_mean7'

mod11 = MultipleGee("11", for11, gee_train)

GEEBinaryClassificationReport(mod11, gee_test, gee_test['acute'],0.2)

GEEBinaryClassificationReport(mod11,gee_valid,gee_valid['acute'],0.2)
