# Supplementary material for: Development of a daily predictive model for the exacerbation of chronic obstructive pulmonary disease
Source: Sci Rep. 2023 Oct 31;13:18669. doi: 10.1038/s41598-023-45835-4 (PMC10618439; doi:10.1038/s41598-023-45835-4)
Supplement: Supplementary file 3 — Supplementary Information 2. [file 41598_2023_45835_MOESM3_ESM.docx]

**"Online Supplementary Methods"**

**Development of a daily predictive model for the exacerbation of chronic obstructive pulmonary disease**

**Supplementary Methods**

**Statistical analysis**

To evaluate the performance of predictive model, we analyzed accuracy which represents the number of correctly classified data instances over the total number of data instances defined as $\frac{true negative \left( TN \right)+true positive (TP)}{total number of data}$, and the area under the curve (AUC) of the receiving operating characteristic curve. Moreover, precision, recall and F1 score were also used as indicators for the possibility of low predictive power of model with very few events of interest and imbalanced dataset. Precision (positive predictive value) is defined as $\frac{TP}{TP+false positive (FP)}$ and should higher and ideally be 1 for a good classifier. Recall is also known as sensitivity or true positive rate and is defined as $\frac{TP}{TP+ false negative (FN)}$. F1 score is a metric which takes into account both precision and recall altogether and is defined as 2$\times$ $\frac{Precision \times Recall}{Precision + Recall}$. F1 score is the harmonic indicator of precision and recall and becomes high only when both precision and recall are high.

To evaluate the contribution of various clinical variables in the predictive model, logistic regression was applied for GEE model and feature importance plot was applied in tree based models. For GEE model, univariate and stepwise multivariate logistic analyses with variables selected by a significance level of entry 0.05 were conducted and data were presented as odds ratio (ORs) and 95% confidence intervals (CI). The threshold (cut-off) value is used to make a binary classification decision based on the probability. If the probability is greater than or equal to the threshold, then the model predicts the positive class or vice versa.

**Prediction equation generated by GEE analysis**

The response vector for subject $i$ is $Y_{i}=\left( y_{i1}, y_{i2},\ldots, y_{in_{i}} \right)$, where $y_{ij}$ is the binary response for subject $i$ and measurement (time point) j.

In the application of GEEs for this analysis, the logit link is specified to indicate the association between the covariates and the binary outcome. The logit model in this context is written as

$$logit\left( p_{ij} \right)=\log\left( \frac{p_{ij}}{1-p_{ij}} \right)=X_{ij}^{'}\beta= \beta_{0}+\beta_{1}*Se{x_{Male}}_{i}+\beta_{2}*Smoking statu{s_{Never}}_{ij}+\beta_{3}*Smoking statu{s_{EX}}_{ij}+\beta_{4}*Smoking statu{s_{Current}}_{ij}+\beta_{5}* BDR FEV1 pcForecas_{ij}+\beta_{6}* Number of times ICS or LABA was taken in 365 \mathrm{days}_{\mathrm{ij}}+\beta_{7}* Number of times LAMA was taken in 90 \mathrm{days}_{\mathrm{ij}}+\beta_{8}*Number of times SABA was taken in 182 \mathrm{days}_{\mathrm{ij}}+\beta_{9}*Number of times OCS was taken in 90days_{ij}+\beta_{10} *Number of hospital visits with anemia in 365 \mathrm{days}_{\mathrm{ij}}+\beta_{11}*Number of hospital visits with COPD in 182 days_{ij}+\beta_{12} Cumulative numbers of \mathrm{AEs}_{\mathrm{ij}}+\beta_{13}Cumulative numbers of severe \mathrm{AEs}_{\mathrm{ij}}+\beta_{14}Number of AEs in 182 \mathrm{days}_{\mathrm{ij}}+\beta_{15}Number of AEs in 90 \mathrm{days}_{\mathrm{ij}}+\beta_{16}Average NO2 value in 42 \mathrm{days}_{\mathrm{ij}}+\beta_{17}Average PM2.5 value in 42 days_{\mathrm{ij}}+\beta_{18}Average humidity for a week_{ij}\boldsymbol{=}\boldsymbol{-}0.871-0.280\boldsymbol{*} Se{x_{Male}}_{i}-1.539* Smoking statu{s_{Never}}_{ij}+0.02* Smoking statu{s_{EX}}_{ij}+0.125* Smoking statu{s_{Current}}_{ij} -0.005* BDR FEV1 pcForecas_{ij}+ 0.03* Number of times ICS, LABA was taken in 365 \mathrm{days}_{\mathrm{ij}}-0.098*Number of times LAMA was taken in 90 \mathrm{days}_{\mathrm{ij}}+0.046* Number of times SABA was taken in 182 \mathrm{days}_{\mathrm{ij}}+0.117* Number of times OCS was taken in 90 days_{ij}-0.132* Number of hospital visits with anemia in 365 \mathrm{days}_{\mathrm{ij}}-0.132* Number of hospital visits with COPD in 182 days_{ij}+ 0.031* Cumulative numbers of \mathrm{AEs}_{\mathrm{ij}}+0.145* Cumulative numbers of severe \mathrm{AEs}_{\mathrm{ij}}+0.228* Number of AEs in 182 \mathrm{days}_{\mathrm{ij}}+0.277* Number of AEs in 90 \mathrm{days}_{\mathrm{ij}}-18.905* Average NO2 value in 42 \mathrm{days}_{\mathrm{ij}}+0.015*Average PM2.5 value in 42 days_{\mathrm{ij}}-0.008* Average humidity for a week_{ij}$$

Where $p_{ij}$is the marginal expectation of the COPD acute exacerbation for subject $i$ at time point j, and $X_{ij}$ is the covariate vector.

**Table S1. Univariate analysis of patient-derived personal factors for the prediction of COPD AE**

| **Variable** | **OR** | **p-value** | **95% CI** | |  |
| --- | --- | --- | --- | --- | --- |
| Male sex | 0.546 | 0.010 | 0.344 - 0.867 | |  |
| Weight | 0.983 | 0.031 | 0.968 – 0.998 | |  |
| FEV_1_ of % predicted value | 0.983 | 0.006 | 0.971 – 0.995 | |  |
| Post-BD FEV_1_ of % predicted value | 0.981 | 0.002 | 0.968 – 0.993 | |  |
| FEV_1_/FVC of % predicted value | 0.982 | 0.083 | 0.962 – 1.002 | |  |
| DLCO of % predicted value | 0.991 | 0.022 | 0.983 – 0.999 | |  |
| Total CAT score | 1.037 | 0.012 | 1.008 – 1.068 | |  |
| Smoking status |  |  |  |  | |
| Ex | 1.123 | 0.519 | 0.790 – 1.597 | |  |
| Current | 1.128 | 0.662 | 0.657 – 1.939 | |  |
| Smoking pack-year | 0.967 | <0.001 | 0.962 – 0.973 | |  |
| Age | 1.012 | 0.320 | 0.988 – 1.036 | |  |
| Number of hospital visits with disease in 365 days |  |  |  |  | |
| Ischemic heart disease | 0.999 | 0.937 | 0.961 - 1.037 | |  |
| Lung cancer | 0.939 | 0.263 | 0.840 - 1.049 | |  |
| Osteoporosis | 1.042 | 0.007 | 1.011 - 1.073 | |  |
| Depression | 0.998 | 0.901 | 0.972 - 1.025 | |  |
| Arthritis | 1.028 | 0.154 | 0.990 - 1.068 | |  |
| Diabetes mellitus | 0.992 | 0.473 | 0.971 - 1.014 | |  |
| Gastroesophageal reflux | 1.029 | 0.012 | 1.006 - 1.051 | |  |
| Congestive heart failure | 1.104 | 0.052 | 0.999 - 1.219 | |  |
| Hypertension | 1.008 | 0.595 | 0.979 - 1.038 | |  |
| Anemia | 0.897 | 0.058 | 0.801 - 1.004 | |  |
| Number of hospital visits with COPD |  |  |  |  | |
| In 365 days | 1.039 | 0.001 | 1.016 - 1.063 | |  |
| In 182 days | 1.058 | 0.003 | 1.019 - 1.098 | |  |
| In 90 days | 1.090 | 0.002 | 1.033 - 1.151 | |  |
| Date since last hospital visit for COPD | 0.998 | 0.194 | 0.996 - 1.001 | |  |
| Visiting referral hospital with a COPD last time | 0.808 | 0.101 | 0.626 - 1.042 | |  |
| Maximum number of days hospital visits due to COPD | 1.083 | <0.001 | 1.049 - 1.119 | |  |
| Maximum number of days of hospital visits and prescription for COPD | 0.998 | 0.288 | 0.995 - 1.002 | |  |
| Cumulative numbers of AEs | 1.087 | <0.001 | 1.061 - 1.114 | |  |
| Cumulative numbers of severe AEs | 1.635 | <0.001 | 1.413 - 1.891 | |  |
| Number of AEs in 365 days | 1.280 | <0.001 | 1.229 - 1.332 | |  |
| Number of AEs in 182 days | 1.557 | <0.001 | 1.465 - 1.655 | |  |
| Number of AEs in 90 days | 2.083 | <0.001 | 1.904 - 2.278 | |  |
| Number of visiting referral hospital with a COPD in 365 days | 1.026 | 0.141 | 0.992 - 1.062 | |  |

AE, acute exacerbation; BD, bronchodilator; CAT, chronic obstructive pulmonary disease (COPD) assessment test; CI, confidence interval; DLCO, diffusing capacity for carbon monoxide; FEV_1_, forced expiratory volume in 1 s; FVC, forced vital capacity

**Table S2. Univariate analysis of respiratory drugs for the prediction of COPD AE.**

| **Variable** | **OR** | **p-value** | **95% CI** | |
| --- | --- | --- | --- | --- |
| **Number of times ICS/LABA was taken** |  |  |  |  |
| In 365 days | 1.054 | 0.014 | 1.011 - 1.100 | |
| In 182 days | 1.104 | 0.007 | 1.028 - 1.186 | |
| In 90 days | 1.237 | <0.001 | 1.099 - 1.392 | |
| **Number of times LABA was taken** |  |  |  |  |
| In 365 days | 1.385 | 0.526 | 0.505 - 3.797 | |
| In 182 days | 1.193 | 0.796 | 0.313 - 4.544 | |
| In 90 days | 1.571 | 0.593 | 0.300 - 8.228 | |
| **Number of times LAMA was taken** |  |  |  |  |
| In 365 days | 0.772 | <0.001 | 0.740 - 0.806 | |
| In 182 days | 0.620 | <0.001 | 0.573 - 0.670 | |
| In 90 days | 0.411 | <0.001 | 0.353 - 0.478 | |
| **Number of times SABA was taken** |  |  |  |  |
| In 365 days | 1.107 | <0.001 | 1.071 - 1.145 | |
| In 182 days | 1.200 | <0.001 | 1.130 - 1.276 | |
| In 90 days | 1.364 | <0.001 | 1.214 - 1.532 | |
| **Number of times SAMA was taken** |  |  |  | |
| In 365 days | 1.184 | <0.001 | 1.111 - 1.261 | |
| In 182 days | 1.348 | <0.001 | 1.229 - 1.478 | |
| In 90 days | 1.753 | <0.001 | 1.518 - 2.024 | |
| **Number of times SABA or SAMA was taken** |  |  |  | |
| In 365 days | 1.112 | 0.339 | 0.895 - 1.381 | |
| In 182 days | 1.199 | 0.339 | 0.826 - 1.740 | |
| In 90 days | 1.464 | 0.307 | 0.705 - 3.037 | |
| **Number of times ICS was taken** |  |  |  | |
| In 365 days | 1.061 | 0.146 | 0.980 - 1.149 | |
| In 182 days | 1.162 | 0.144 | 0.950 - 1.422 | |
| In 90 days | 1.261 | 0.088 | 0.966 - 1.646 | |
| **Number of times OCS was taken** |  |  |  | |
| In 365 days | 1.137 | <0.001 | 1.111 - 1.164 | |
| In 182 days | 1.269 | <0.001 | 1.220 - 1.321 | |
| In 90 days | 1.499 | <0.001 | 1.395 - 1.611 | |

CI, confidence interval; ICS, inhaled corticosteroid; LABA, long-acting beta2 receptor agonist; LAMA, long-acting muscarinic receptor antagonist; OCS, oral corticosteroids; OR, odds ratio; SABA, short-acting beta2 receptor agonist; SAMA, short-acting muscarinic receptor antagonist

**Table S3. Univariate analysis of environmental factors for the prediction of COPD AE.**

| **Variable** | **OR** | **p-value** | **95% CI** |
| --- | --- | --- | --- |
| Average PM_10_ value for a week | 1.000 | 0.919 | 0.997 - 1.003 |
| Maximum of PM_10_ value for a week | 1.000 | 0.688 | 0.999 - 1.001 |
| Average PM_10_ value in 42 days | 1.000 | 0.945 | 0.995 - 1.005 |
| Average of NO_2_ value for a week | 0.000 | 0.017 | 0.000 - 0.096 |
| Maximum of NO_2_ value for a week | 0.000 | 0.017 | 0.000 - 0.201 |
| Average NO_2_ value in 42 days | 0.000 | 0.016 | 0.000 - 0.058 |
| Average PM_2.5_ value for a week | 0.946 | <0.001 | 0.940 - 0.952 |
| Maximum of PM_2.5_ value for a week | 0.966 | <0.001 | 0.962 - 0.970 |
| Average PM_2.5_ value in 42 days | 0.945 | <0.001 | 0.939 - 0.950 |
| Average temperature for a week | 0.994 | 0.043 | 0.988 - 1.000 |
| Maximum daily temperature difference for a week | 1.027 | 0.085 | 0.996 - 1.059 |
| Average humidity for a week | 0.994 | 0.058 | 0.988 - 1.000 |
| Amount of IFV detection | 1.001 | 0.001 | 1.001 - 1.002 |
| IFV detection rate | 1.006 | <0.001 | 1.003 - 1.010 |

IFV, influenza virus; NO_2_, nitrogen dioxide; PM_2.5_, particulate matter (PM) with aerodynamic diameters ≤ 2.5 μm; PM_10_, PM with aero­dynamic diameters ≤ 10 μm

**Table S4. Prediction performance in internal validation cohort (KOCOSS)**

|  | Mean | 95% CI Lower limit | 95% CI upper limit |
| --- | --- | --- | --- |
| Random Forest |  |  |  |
| AUC | 0.913 | 0.904 | 0.922 |
| Accuracy | 0.816 | 0.808 | 0.825 |
| F1 score | 0.588 | 0.573 | 0.604 |
| Sensitivity (Recall) | 0.860 | 0.838 | 0.879 |
| PPV (Precision) | 0.447 | 0.434 | 0.462 |
| Specificity | 0.808 | 0.798 | 0.820 |
| NPV | 0.970 | 0.965 | 0.974 |
| Cut-off value | 0.3 |  |  |
| XGBoost |  |  |  |
| AUC | 0.925 | 0.917 | 0.931 |
| Accuracy | 0.874 | 0.864 | 0.883 |
| F1 score | 0.644 | 0.624 | 0.665 |
| Sensitivity (Recall) | 0.745 | 0.720 | 0.766 |
| PPV (Precision) | 0.568 | 0.540 | 0.592 |
| Specificity | 0.898 | 0.886 | 0.908 |
| NPV | 0.951 | 0.947 | 0.955 |
| Cut-off value | 0.3 |  |  |
| LGBM |  |  |  |
| AUC | 0.936 | 0.930 | 0.942 |
| Accuracy | 0.869 | 0.858 | 0.878 |
| F1 score | 0.656 | 0.638 | 0.672 |
| Sensitivity (Recall) | 0.818 | 0.796 | 0.837 |
| PPV (Precision) | 0.548 | 0.523 | 0.569 |
| Specificity | 0.878 | 0.866 | 0.886 |
| NPV | 0.964 | 0.960 | 0.968 |
| Cut-off value | 0.211 |  |  |
| MERF |  |  |  |
| AUC | 0.914 | 0.904 | 0.923 |
| Accuracy | 0.845 | 0.835 | 0.855 |
| F1 score | 0.611 | 0.592 | 0.633 |
| Sensitivity (Recall) | 0.797 | 0.767 | 0.823 |
| PPV (Precision) | 0.495 | 0.476 | 0.517 |
| Specificity | 0.853 | 0.843 | 0.863 |
| NPV | 0.959 | 0.953 | 0.964 |
| Cut-off value | 0.22 |  |  |
| Logistic |  |  |  |
| AUC | 0.802 | 0.786 | 0.817 |
| Accuracy | 0.780 | 0.770 | 0.790 |
| F1 score | 0.489 | 0.473 | 0.509 |
| Sensitivity (Recall) | 0.690 | 0.660 | 0.723 |
| PPV (Precision) | 0.379 | 0.363 | 0.396 |
| Specificity | 0.796 | 0.783 | 0.806 |
| NPV | 0.934 | 0.929 | 0.941 |
| Cut-off value | 0.5 |  |  |
| SVM |  |  |  |
| AUC | 0.736 | 0.721 | 0.755 |
| Accuracy | 0.773 | 0.764 | 0.789 |
| F1 score | 0.479 | 0.460 | 0.503 |
| Sensitivity (Recall) | 0.683 | 0.648 | 0.712 |
| PPV (Precision) | 0.369 | 0.354 | 0.392 |
| Specificity | 0.789 | 0.778 | 0.805 |
| NPV | 0.932 | 0.926 | 0.939 |
| Cut-off value | - |  |  |
| GEE |  |  |  |
| AUC | 0.822 | 0.802 | 0.840 |
| Accuracy | 0.846 | 0.836 | 0.853 |
| F1 score | 0.522 | 0.495 | 0.542 |
| Sensitivity (Recall) | 0.552 | 0.524 | 0.578 |
| PPV (Precision) | 0.495 | 0.467 | 0.517 |
| Specificity | 0.899 | 0.891 | 0.907 |
| NPV | 0.918 | 0.912 | 0.922 |
| Cut-off value | 0.2 |  |  |

AUC, area under the curve; GEE, generalized estimating equation; KOCOSS, the Korean COPD subgroup study; LGBM, light gradient boosted machine; MERF, mixed effect random forest; NPV, negative predicted value; PPV, positive predictive value; SVM, support vector machine; XGBoost, extreme gradient boosting

**Table S5. The number of exacerbations before and after resampling in the training and validation data**

|  | **Training cohort**  **(before resampling)** | **Test cohort**  **(after resampling)** | **Internal validation cohort** | **External validation cohort** |
| --- | --- | --- | --- | --- |
| Cases | 12,693 | 21,445 | 5,252 | 9,687 |
| Number of AEs | 1,942 (15.30%) | 10,694 (49.87%) | 955 (15.28%) | 3,639 (37.57%) |

AE, acute exacerbation

**Figure S1. Example scenarios within the testing data set model for prediction of exacerbation**

Exacerbation risk is divided into four quartiles: good, moderate, unhealthy, and very unhealthy. These quartiles are presented by class A to D based on the risk of occurrence of exacerbation. For instance, in Scenario A, given specific data including climate conditions, air quality and virus detection rates on a particular day, if a 52-year-old non-smoking male patient with COPD, possessing a FEV_1_ of 75% of predicted value, no history of smoking, using LAMA, and a year without prior exacerbation, experiences an exacerbation, the calculated risk stands at 11%. Thus, this risk assessment places the patient in Class A. In Scenario B, within a defined set of weather conditions, air quality parameters, and virus detection rates, patient who is 74 years old, currently smoking, using ICS/LABA/LAMA therapy, has an FEV_1_ of 35% of predicted value, and experienced three exacerbations within the preceding year. The calculated risk for exacerbation is 97%, thereby warranting classification as Class D.

AE, acute exacerbation; FEV_1_, forced expiratory volume in 1 s; ICS, inhaled corticosteroid; LABA, long-acting beta2 receptor agonist; LAMA, long-acting muscarinic receptor antagonist; NO_2_, nitrogen dioxide; PM, particulate matter
